# Murine vs. Human Osteoblast Responses to Coagulation and Inflammatory Factors: Reconsidering the Use of Animal Models in Hemophilia A Research

**DOI:** 10.3390/biomedicines12122666

**Published:** 2024-11-22

**Authors:** Aline Bernar, Monika Bauer, Michael Schirmer, Werner Streif, Jennifer Gebetsberger

**Affiliations:** 1 Department of Pediatrics I, Medical University Innsbruck, 6020 Innsbruck, Austria; aline.bernar@i-med.ac.at (A.B.); werner.streif@i-med.ac.at (W.S.); 2 Department of Internal Medicine II, Medical University Innsbruck, 6020 Innsbruck, Austria; moni.bauer@tirol-kliniken.at (M.B.); schirmer.michael@icloud.com (M.S.)

**Keywords:** mice, humans, osteoblasts, coagulation factors, cytokines, bone mineralization, species specificity, hemophilia A

## Abstract

**Background/Objectives**: Hemophilia A is associated with frequent bleeding episodes, joint damage, and reduced bone mineral density (BMD). The role of coagulation factors and inflammatory cytokines on bone metabolism, particularly on osteoblast function, is of increasing interest. However, significant inter-species differences in bone remodeling raise concerns about the translatability of findings from murine models to human systems. This study aims to investigate the effects of human coagulation factors and cytokines on bone formation, focusing on inter-species differences in the cell viability and mineralization of murine and human osteoblasts. **Methods**: Murine MC3T3-E1 and human SaOs-2 osteoblasts were cultured in osteoblast differentiation medium supplemented with various coagulation factors (FVIII, vWF, vWF-FVIII, FIX, FX, thrombin, and FVIII-thrombin) or cytokines (IL-6, TNF-α). Cell viability was assessed at both two-week and three-week time points using the CCK-8 assay, and mineralization was evaluated via Alizarin red S staining. **Results:** Coagulation factors significantly enhanced cell viability in human osteoblasts but had no effects on the murine counterpart. FX inhibited mineralization in human cells, while murine cells showed no significant changes. TNF-α stimulated mineralization in murine osteoblasts but inhibited it in human cells, highlighting species-specific responses to inflammatory cytokines. Similar trends in response patterns were observed at two and three weeks, with greater consistency at the later time point. **Conclusions**: These findings reveal critical inter-species differences in osteoblast responses to coagulation factors and cytokines, raising questions about the validity of using murine models to study human bone metabolism. Future research must account for these differences to ensure that preclinical models accurately reflect human pathophysiology, particularly in the context of hemophilia A.

## 1. Introduction

Hemophilia A is a genetic disorder characterized by a deficiency in coagulation factor VIII (FVIII), resulting in frequent bleeding episodes, joint damage, and a well-documented risk of reduced bone mineral density (BMD) [1]. Studies have shown that up to 70% of hemophilia A patients are affected by low BMD, leading to diagnoses of osteopenia and osteoporosis [2]. This bone loss is not solely a consequence of joint damage due to recurrent hemarthrosis, but is also associated with systemic disruptions in bone metabolism, linked directly to the underlying coagulopathy [1,3].

Osteoblasts, the primary cells responsible for bone formation, are affected by both coagulation factors and inflammatory cytokines. Coagulation factors such as thrombin and factor X (FX) play important roles in regulating osteoblast proliferation, differentiation, and matrix mineralization [4,5,6,7,8]. In addition, frequent bleeding episodes often trigger inflammatory responses, leading to the release of cytokines like tumor necrosis factor-alpha (TNF-α) and interleukin-6 (IL-6) [9,10]. These cytokines not only contribute to bone resorption but also further impair osteoblast function, ultimately leading to bone loss [9]. These biochemical signals are crucial for understanding bone health in hemophilia A patients, where the combined effects of inflammation and bleeding episodes create a unique environment for bone remodeling.

However, most of the research investigating the relationship between coagulation factors, cytokines, and bone metabolism has been conducted using murine models [11,12,13,14,15,16]. Although murine models are invaluable in scientific research due to their genetic manipulability and easy handling [17,18], significant species-specific differences may limit their translational relevance. For instance, murine osteoblasts may exhibit different responses to human coagulation factors such as FVIII and FX compared to human osteoblasts, potentially due to variations in receptor expression and intracellular signaling pathways [17]. Previous studies, for example, have highlighted the importance of calcium signaling in osteoblasts for matrix mineralization [19,20], and how disruptions in this pathway could differ between human and murine cells when exposed to the same factors [7,21]. Furthermore, murine platelets lack protease-activated receptor 1 (PAR-1) [22], a critical receptor for thrombin signaling in humans [23], which at least partly explains why thrombin could affect osteoblast activity in mice differently than in humans. Additionally, murine models of hemophilia do not naturally experience spontaneous joint bleeds—a hallmark of human hemophilia—making it difficult to fully capture the consequences of chronic hemarthrosis on bone health [24,25]. These species-specific responses may lead to underestimations of the impact of coagulation factors and cytokines on human bone health when relying solely on murine models.

Given the limitations of murine models and the fact that several human-modified coagulation factors, such as B-domain deleted FVIII, used in research and clinical applications, lack direct murine equivalents, it is crucial to directly compare the effects of human coagulation factors and cytokines at least on both human and murine osteoblasts. The present study aims to investigate interspecies differences in osteoblast responses to coagulation factors (FVIII, vWF, vWF-FVIII, FIX, FX, thrombin and FVIII-thrombin) and inflammatory cytokines (IL-6 and TNF-α) to better understand the translational limitations of murine models. By examining cell viability and mineralization at both two-week and three-week time points in both murine (MC3T3-E1) and human (SaOs-2) osteoblasts, two commonly used cell lines in osteoblast research [26], we seek to provide species-specific insights into the challenges of transforming murine data to human clinical contexts, particularly for deeper understanding of bone remodeling in hemophilia A patients.

## 2. Materials and Methods

### 2.1. Cell Lines and Growth Conditions

Depending on the experiment, either the murine MC3T3-E1 or human SaOs-2 cell line was used. These cell lines were selected for their established use in osteoblast research, offering advantages such as better accessibility, ease of maintenance, and consistency in experimental outcomes. Unlike primary cells, which can exhibit donor-related phenotypic variations, these cell lines provide a more uniform model, suitable for long-term experiments. Both cell lines were cultured at 37 °C with 5% CO_2_ in α-minimum essential medium (αMEM; GibcoTM, Thermo Fisher Scientific, Waltham, MA, USA) supplemented with 10% fetal bovine serum (GibcoTM, Thermo Fisher Scientific, Waltham, MA, USA; same batch) and 1% Penicillin–Streptomycin (GibcoTM, Thermo Fisher Scientific, Waltham, MA, USA). The medium was supplemented with 1 mM ascorbic acid (Merck KGaA, Darmstadt, Germany), 8 mM β-glycerophosphate (Merck KGaA, Darmstadt, Germany), and 5 mM calcium chloride (Merck KGaA, Darmstadt, Germany) to induce mineralization, as described by Bernar et al., 2022 [27]. This supplemented medium is referred to as osteoblast differentiation medium (ODM+). The medium was changed weekly in all experiments.

### 2.2. Coagulation Factors and Cytokines

Human coagulation factor VIII (ADVATE, Takeda Pharmaceutical, Tokyo, Japan), vWF (VEYVONDI, Takeda Pharmaceutical, Tokyo, Japan), vWF-FVIII (Haemoctin SDH, Biotest AG, Dreieich, Germany), FIX (BeneFix, Pfizer, New York City, NY, USA), and FX (Factor XP Behring, CSL Behring, King of Prussia, PA, USA) were all kindly provided by the companies as mentioned. Thrombin from human plasma was purchased from Merck KGaA, Darmstadt, Germany. IL-6 and TNF-α were obtained from PeproTech (Thermo Fisher Scientific, Waltham, MA, USA).

All coagulation factors were reconstituted using a 1:2 (*v*/*v*) ratio of water for injection (WFI) and glycerol (Merck KGaA, Darmstadt, Germany), resulting in a final concentration of 25 U/mL. IL-6 and TNF-α were dissolved according to the manufacturers’ instructions. Short, IL-6, and TNF-α were reconstituted in water to a final concentration of 10 µg/mL. A total of 0.1% BSA (Merck KGaA, Darmstadt, Germany) was added to both solutions to stabilize the substances during storage.

### 2.3. Experimental Setup

To analyze the effects of various coagulation factors and cytokines on osteoblasts, different passages of murine MC3T3-E1 and human SaOs-2 osteoblastic cells were seeded in 96-well plates at a density of 6500 cells/well. After 24 h of incubation, the 100 µL medium was replaced with 100 µL ODM+ containing different coagulation factors (FVIII, vWF, vWF-FVIII, FIX, FX, thrombin, and FVIII-thrombin; 1 U/mL each) or cytokines (IL-6 and TNF-α; 50 ng/mL each). The negative control consisted of α-MEM medium without any supplements (ODM−). The medium was changed weekly. After two or three weeks of incubation at 37 °C with 5% CO_2_, cell viability and mineralization were assessed. All results are presented as the means ± standard deviations (SD) from at least four independent experiments with biological triplicates.

For the experiments, human SaOs-2 cells were obtained from a single frozen vial to ensure consistency in the initial cell population, while murine MC3T3-E1 cells were derived from two frozen vials of the same origin. Each experiment used cells at different passages, with separate cell populations seeded into each well to ensure biological replicates. No repeated measurements were taken from the same sample, and each experiment was initiated on a different day to ensure independence. Additionally, cell passages were carefully monitored to prevent the use of cells at stages where mineralization or viability could be compromised.

### 2.4. Cell Viability

Cell viability was assessed using the Cell Counting Kit-8 (CCK-8) (Merck KGaA, Darmstadt, Germany). Briefly, 20 µL of the diluted CCK-8 solution in α-MEM medium was added to each well directly to the culture medium without removing any residual medium including the coagulation factors or cytokines. After two hours of incubation at 37 °C with 5% CO_2_, absorbance at 450 nm was measured using a microplate reader (Tecan Spark Multimode Microplate Reader, Tecan Group, Männedorf, Switzerland).

### 2.5. Alizarin Red S Staining and Photometric Quantification

Alizarin red S staining and photometric quantification were performed as previously described [27].

### 2.6. Statistical Analysis

All results are presented as the means ± standard deviations (SD) from at least four independent experiments with biological triplicates. Differences between groups were assessed using a one-way ANOVA on log-transformed data followed by a post hoc test using Dunnett’s method. *p*-values < 0.05 were considered statistically significant.

## 3. Results

### 3.1. Cell Viability of Murine MC3T3-E1 and Human SaOs-2 Osteoblasts After Stimulation with Different Coagulation Factors

**Figure 1:**
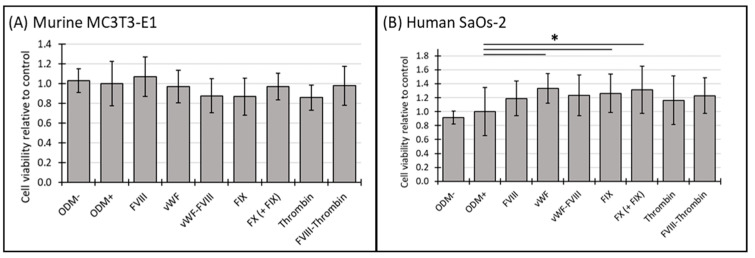
The cell viability of murine MC3T3-E1 and human SaOs-2 osteoblasts in the presence of various coagulation factors. (**A**) Murine MC3T3-E1 osteoblasts and (**B**) human SaOs-2 osteoblasts were incubated for three weeks in αMEM medium supplemented with ascorbic acid (1 mM), ß-glycerophosphate (8 mM), and calcium chloride (5 mM), referred to as osteoblast differentiation medium (ODM+). The negative control (ODM−) lacked these supplements. Cells were exposed to various coagulation factors (FVIII, vWF, vWF-FVIII, FIX, FX, thrombin, and FVIII-thrombin; 1 U/mL each). Cell viability was assessed relative to the ODM+ reference, set as 1. Data represent the means ± standard deviation of four independent assays performed in triplicate (n = 3), except for ODM+ reference and the negative control (ODM−) (n = 6). * indicates a significant difference (*p* < 0.05).

The cell viability of murine MC3T3-E1 osteoblasts and human SaOs-2 osteoblasts was assessed at both two-week and three-week time points to explore temporal effects. In the ODM− control and the positive reference (ODM+), no significant differences in cell viability were observed between murine and human osteoblasts (Figure 1A,B). Among the coagulation factors tested, only FVIII-vWF showed a significant reduction in cell viability of murine MC3T3-E1 osteoblasts after two weeks (Supplementary Figure S1A), an effect that was no longer observed after three weeks (Figure 1A).

In contrast, the presence of the coagulation factors in the ODM+ medium of SaOs-2 osteoblasts enhanced cell viability after both two and three weeks. Specifically, after three weeks vWF, FIX and FX increased cell viability by 33%, 26% and 31%, respectively. The observed increases were as follows: vWF from 1.0 to 1.33 ± 0.21 (*p* = 0.002), FIX from 1.0 to 1.26 ± 0.28 (*p* = 0.024), and FX from 1.0 to 1.31 ± 0.34 (*p* = 0.011) (Figure 1B). Similar trends were noted at the 2-week time point, where FX and the combination FX(+FIX) also significantly increased cell viability (Supplementary Figure S1B), suggesting a consistent response over time in the human osteoblasts.

Four independent assays using triplicates, except for the positive reference and the negative control (n = 6), were performed. Cell viability was assessed using the CCK-8 assay, as described in the Section 2. For quantification and comparison, the ODM+ reference was set as value 1.

### 3.2. Mineralization of Murine MC3T3-E1 and Human SaOs-2 Osteoblasts After Stimulation with Different Coagulation Factors

Following the assessment of cell viability, the functionality of murine MC3T3-E1 and human SaOs-2 osteoblasts was further evaluated using a mineralization assay. Mineralization was assessed at both two-week and three-week time points to capture temporal effects.

**Figure 2:**
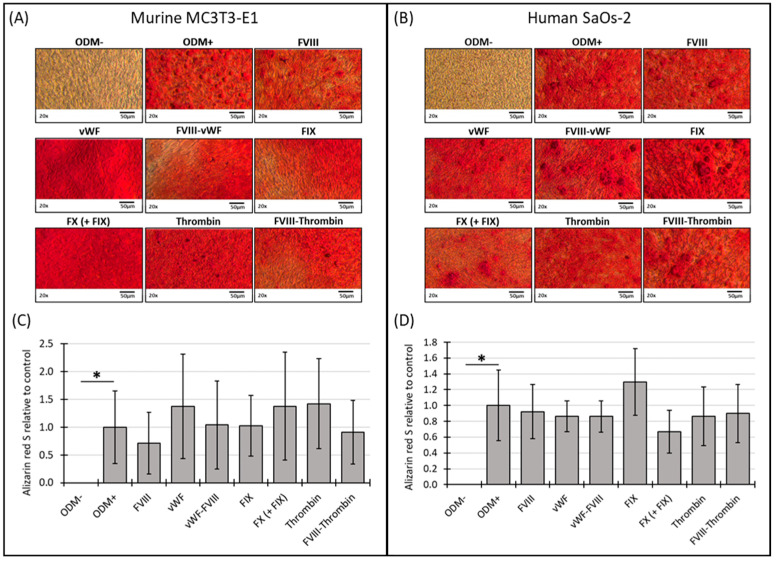
Representative images and photometric quantification of Alizarin red S staining in murine MC3T3-E1 and human SaOs-2 osteoblasts. Representative images of (**A**) murine MC3T3-E1 osteoblasts and (**B**) human SaOs-2 osteoblasts after three weeks of incubation in αMEM medium supplemented with ascorbic acid (1 mM), ß-glycerophosphate (8 mM), and calcium chloride (5 mM), referred to as osteoblast differentiation medium (ODM+). The negative control (ODM−) lacked these supplements. Cells were exposed to different coagulation factors (FVIII, vWF, vWF-FVIII, FIX, FX, thrombin, and FVIII-thrombin; 1 U/mL each). After incubation, cells were stained with Alizarin red S to assess mineralization, which was then quantified photometrically. Quantification results are shown for (**C**) murine and (**D**) human osteoblasts, with data normalized to the ODM+ reference. Data are presented as the means ± standard deviation of four independent assays performed in triplicate (n = 3), except for the ODM+ reference and the negative control (ODM−) (n = 6). * indicates a significant difference (*p* < 0.05). Images were captured using transmitted light microscopy at 20× magnification. Scale bar: 50 µm.

No mineralization was observed in MC3T3-E1 osteoblasts when cultured in α-MEM medium without differentiation factors (ODM−). However, when differentiation factors were present in the ODM+ medium, mineralization became visible by the bright red stain on the images (Figure 2A) and detectable photometrically (Figure 2C). After two weeks, the Alizarin red S staining of murine MC3T3-E1 osteoblasts was relatively weak, likely due to the cells not yet reaching full maturation, leading to high variability and standard deviations in the measurements (Supplementary Figure S2A,C). Consequently, these early data are challenging to interpret definitively. By three weeks, the mineralization in the murine osteoblasts appeared more consistent, with the addition of the coagulation factors to the ODM+ medium not significantly altering mineralization compared to the ODM+ reference (Figure 2A,C).

Similar, in human SaOs-2 osteoblasts, while differentiation factors induced mineralization (ODM+), as seen on the representative images of the staining (Figure 2B), the presence of the coagulation factors did not induce a significant difference in mineralization compared to the positive reference at either two or three weeks (Supplementary Figure S2B,D and Figure 2B,D). These findings suggest that, particularly in murine cells, mineralization stabilizes between two and three weeks, while the addition of coagulation factors does not significantly impact this process in either murine or human osteoblasts under the conditions tested.

### 3.3. Mineralization Relative to Cell Viability of Murine MC3T3-E1 and Human SaOs-2 Osteoblasts After Stimulation with Different Coagulation Factors

**Figure 3:**
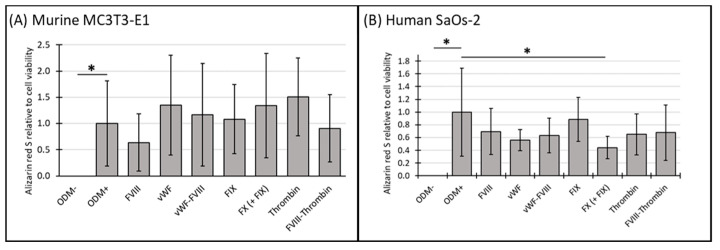
The expression of Alizarin red S relative to cell viability in murine MC3T3-E1 and human SaOs-2 osteoblasts. (**A**) Murine MC3T3-E1 osteoblasts and (**B**) human SaOs-2 osteoblasts were incubated for three weeks in αMEM medium supplemented with ascorbic acid (1 mM), ß-glycerophosphate (8 mM), and calcium chloride (5 mM), referred to as osteoblast differentiation medium (ODM+). The negative control (ODM−) lacked these supplements. Cells were exposed to various coagulation factors (FVIII, vWF, vWF-FVIII, FIX, FX, thrombin, and FVIII-thrombin; 1 U/mL each). The levels of Alizarin red S were quantified relative to cell viability and are expressed relative to the ODM+ reference, which was set as 1. Data represent the means ± standard deviation of four independent assays performed in triplicate (n = 3), except for the ODM+ reference and the negative control (ODM−) (n = 6). * indicates a significant difference (*p* < 0.05).

To further elucidate the relationship between cell viability and cellular function, we analyzed mineralization relative to cell viability using the data obtained from the previous experiments (see Section 3.1 and Section 3.2). For comparison, the ODM+ reference was normalized to a value of 1. No change in mineralization relative to cell viability was observed in either murine or human osteoblasts when differentiation factors were absent from the α-MEM medium (ODM−). However, the addition of differentiation factors significantly increased mineralization relative to cell viability in both cell lines (Figure 3A,B).

In murine osteoblasts, the presence of coagulation factors in the osteoblast differentiation medium did not significantly alter mineralization relative to cell viability compared to the ODM+ reference at either time point (Figure 3A and Supplementary Figure S3A).

In contrast, in human SaOs-2 osteoblasts, coagulation factor FX significantly reduced mineralization relative to cell viability by 63% at two weeks (from 1.0 to 0.37 ± 0.15, *p* < 0.001) (Supplementary Figure S3B) and by 56% (from 1.0 to 0.44 ± 0.18, *p* = 0.003) (Figure 3B) at three weeks. These findings suggest a consistent inhibitory effect of FX on mineralization in human osteoblasts.

### 3.4. Cell Viability of Murine MC3T3-E1 and Human SaOs-2 Osteoblasts After Stimulation with Cytokines

**Figure 4:**
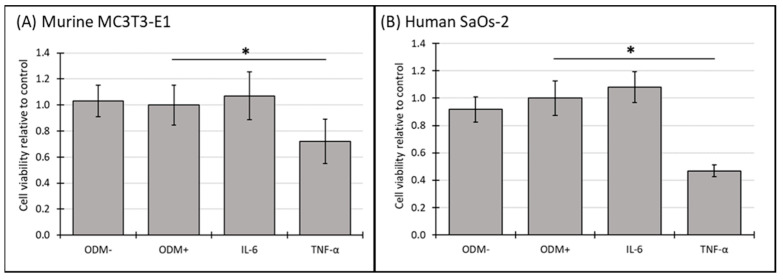
The cell viability of murine MC3T3-E1 and human SaOs-2 osteoblasts in the presence of different cytokines. (**A**) Murine MC3T3-E1 osteoblasts and (**B**) human SaOs-2 osteoblasts were incubated for three weeks in αMEM medium supplemented with ascorbic acid (1 mM), ß-glycerophosphate (8 mM), and calcium chloride (5 mM), referred to as osteoblast differentiation medium (ODM+). The negative control (ODM−) lacked these supplements. Cells were exposed to different cytokines (IL-6 and TNF-α; 50 ng/mL each). Cell viability was assessed relative to the ODM+ reference, which was set as 1. Data represent the means ± standard deviation of four independent assays performed in triplicate (n = 3), except for ODM+ reference and the negative control (ODM−) (n = 6). * indicates a significant difference (*p* < 0.05).

In murine MC3T3-E1 osteoblasts, IL-6 showed a significant reduction in cell viability after two weeks (Supplementary Figure S4A), an effect that was no longer observed at three weeks (Figure 4A). In contrast, TNF-α consistently reduced cell viability across both time points, with a significant decrease of 28% (from 1.0 to 0.72 ± 0.28; *p* = 0.002) (Supplementary Figure S4A) after two weeks and 28% (from 1.0 to 0.72 ± 0.17; *p* < 0.001) (Figure 4A) after 3 weeks.

In human SaOs-2 osteoblasts, the addition of IL-6 did not significantly alter cell viability compared to the ODM+ reference at either time point (Figure 4B and Supplementary Figure S4B). However, similar to its effect on murine osteoblasts, TNF-α significantly decreased cell viability in human osteoblasts, with a reduction of 40% (from 1.0 to 0.60 ± 0.13; *p* < 0.001) (Supplementary Figure S4B) after two weeks and 53% (from 1.0 to 0.47 ± 0.04; *p* < 0.001) (Figure 4B) after three weeks. These results indicate that TNF-α has a stable inhibitory effect on cell viability in both murine and human osteoblasts, while the effect of IL-6 appears transient and is limited to murine osteoblasts at earlier time points.

### 3.5. Mineralization of Murine MC3T3-E1 and Human SaOs-2 Osteoblasts After Stimulation with Cytokines

**Figure 5:**
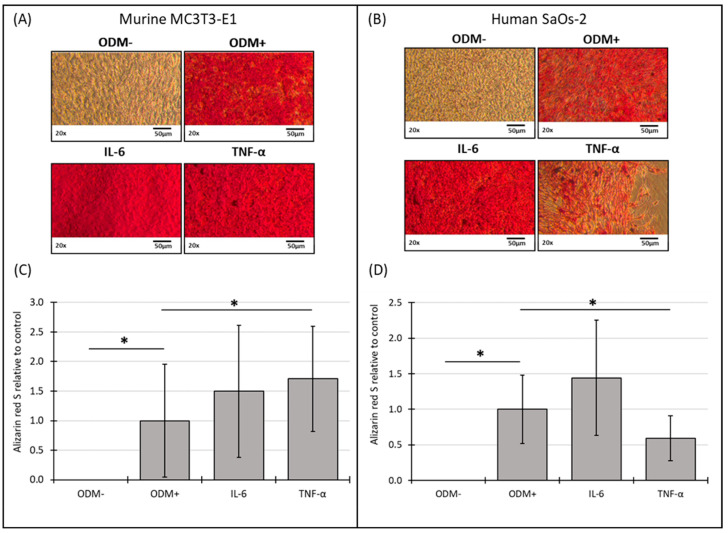
Representative images and photometric quantification of Alizarin red S staining in murine MC3T3-E1 and human SaOs-2 osteoblasts. Representative images of (**A**) murine MC3T3-E1 osteoblasts and (**B**) human SaOs-2 osteoblasts after three weeks of incubation in αMEM medium supplemented with ascorbic acid (1 mM), β-glycerophosphate (8 mM), and calcium chloride (5 mM), referred to as osteoblast differentiation medium (ODM+). The negative control (ODM−) lacked these supplements. Cells were exposed to different cytokines (IL-6 and TNF-α; 50 ng/mL each). After incubation, cells were stained with Alizarin red S to assess mineralization, which was then quantified photometrically. Quantification results are shown for (**C**) murine and (**D**) human osteoblasts, with data normalized to the ODM+ reference. Data are presented as the means ± standard deviation of four independent assays performed in triplicate (n = 3), except for the ODM+ reference and the negative control (ODM−) (n = 6). * indicates a significant difference (*p* < 0.05). Images were captured using transmitted light microscopy at 20× magnification. Scale bar: 50 µm.

The addition of IL-6 did not significantly affect mineralization in either murine or human osteoblasts at either time point compared to the ODM+ reference (Figure 5 and Figure S5). However, TNF-α showed a consistent effect on mineralization in both cell types. In murine osteoblasts, TNF-α significantly induced a 70% increase in mineralization in murine osteoblasts (from 1.0 to 1.7 ± 0.89; *p* = 0.030) (Figure 5C), a trend that was also observed at two weeks but with higher variability (Supplementary Figure S5C). Conversely, in human osteoblasts, TNF-α decreased mineralization by 38% (from 1.0 to 0.62 ± 0.32; *p* = 0.009) after two weeks (Supplementary Figure S5D) and 40% (from 1.0 to 0.6 ± 0.32; *p* = 0.008) after three weeks (Figure 5D).

No mineralization occurred without differentiation factors (ODM−). The presence of differentiation factors in the ODM+ medium induced the mineralization of both cell types (Figure 5A,B). The microscopic results were confirmed by the quantitative analysis of the Alizarin red S staining for murine (Figure 5C, Supplementary Figure S5C) and human osteoblasts (Figure 5D, Supplementary Figure S5D).

### 3.6. Mineralization Relative to Cell Viability of Murine MC3T3-E1 and Human SaOs-2 Osteoblasts After Stimulation with Cytokines

Using the data from Section 3.4 and Section 3.5, the effects of cytokines on mineralization relative to cell viability in both murine MC3T3-E1 and human SaOs-2 osteoblasts were evaluated to further explore the relationship between cell viability in cellular function.

**Figure 6:**
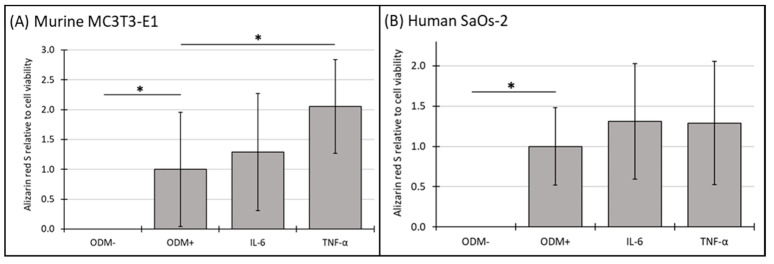
The expression of Alizarin red S relative to cell viability in murine MC3T3-E1 and human SaOs-2 osteoblasts. (**A**) Murine MC3T3-E1 osteoblasts and (**B**) human SaOs-2 osteoblasts were incubated for three weeks in αMEM medium supplemented with ascorbic acid (1 mM), ß-glycerophosphate (8 mM), and calcium chloride (5 mM), referred to as osteoblast differentiation medium (ODM+). The negative control (ODM−) lacked these supplements. Cells were exposed to different cytokines (IL-6 and TNF-α; 50 ng/mL each). The levels of Alizarin red S were quantified relative to the cell viability and are expressed relative to the ODM+ reference, which was set as 1. Values represent the means ± standard deviation of four independent assays performed in triplicate (n = 3), except for ODM+ reference and the negative control (ODM−) (n = 6). * indicates a significant difference (*p* < 0.05).

No mineralization relative to cell viability was observed in the absence of differentiation factors (ODM−). The addition of IL-6 to the medium did not result in significant changes in mineralization relative to cell viability at either time point in either murine (Figure 6A and Supplementary Figure S6A) or human osteoblasts (Figure 6B and Supplementary Figure S6B). However, the presence of TNF-α significantly increased mineralization relative to cell viability by 105% in murine MC3T3-E1 osteoblasts at three weeks, rising from 1.0 to 2.05 ± 0.79 (*p* < 0.006) (Figure 6A), a trend also observed at two weeks but with higher variability (Supplementary Figure S6A). In contrast, TNF-α had no significant impact on this parameter in human SaOs-2 osteoblasts (Figure 6B), indicating a distinct species-specific response.

## 4. Discussion

This study clearly showed interspecies differences between human and murine osteoblast responses to human coagulation factors and inflammatory cytokines, particularly focusing on cell viability and mineralization. The selection of the murine MC3T3-E1 and human SaOs-2 cell lines was strategic due to their widespread use and well-characterized behavior in osteoblast research. These cell lines provide several advantages over primary cells, such as easier access, simpler maintenance, and greater reliability, particularly when conducting multiple experimental replicates. Unlike primary cells, which can exhibit variability due to donor-specific differences, established cell lines offer a more consistent and reproducible model system. MC3T3-E1 cells were chosen for their homogeneity and reliable differentiation potential, making them a preferred model for murine osteoblast studies. Similarly, SaOs-2 cells were selected for their human origin, their capacity for matrix mineralization, and their ability to mitigate interspecies differences [26]. The use of these cell lines helped minimize experimental variability and ensured consistency across long-term studies, which was crucial for assessing the effects of coagulation factors and cytokines on osteoblast function over several weeks.

These findings underscore the challenges of using murine models to study bone health in hemophilia A.

### 4.1. Coagulation Factors and Species-Specific Responses

The data revealed clear species-specific responses to coagulation factors. In murine osteoblasts, none of the coagulation factors altered cell viability. In contrast, human osteoblasts were more responsive to certain coagulation factors, with significant increases in viability observed in response to vWF, FIX, and FX. These findings suggest that murine models may not fully capture the role of coagulation factors on osteoblast viability, emphasizing the limitations of translating findings directly from murine to human systems.

The distinct responses of murine osteoblasts may be explained by species-specific molecular mechanisms, such as differences in the expression of receptors and their associated signaling pathways. For example, while murine and human osteoblasts both express a functional thrombin receptor, PAR-1 [6,8], murine platelets notably lack PAR-1 [22]—a critical receptor for thrombin, but also FX signaling in humans [23]. This absence of PAR-1 in murine platelets may result in different coagulation-mediated effects in mice models compared to humans, which should be considered when using a murine hemophilia A model. Importantly, the cellular effects of coagulation factors are mediated not only by PAR-1, but also by other protease-activated receptors [23]. Differences in how these receptors and their downstream signaling pathways are activated in murine versus human cells could explain divergent responses to coagulation factors.

Additionally, murine FVIII and FX, for example, exhibit only partial homology with their human counterparts, which could impact how coagulation factors interact with osteoblasts across species [28]. These molecular differences indicate that murine models appear to underestimate the impact of coagulation factors on human bone metabolism, especially in hemophilia A, where these factors are chronically dysregulated.

### 4.2. Coagulation Factors and Mineralization

In terms of mineralization, murine osteoblasts showed no significant changes in mineralization when exposed to coagulation factors, whereas human osteoblasts exhibited inhibited mineralization relative to cell viability in response to FX. The role of FX in calcium signaling and osteoblast mineralization has been well-documented in human studies [4], but our findings suggest that murine cells may not rely on the same pathways for bone-matrix deposition. Human osteoblasts may have more complex regulatory mechanisms involving calcium signaling, which are essential for the initiation of mineralization [19]. Effects that are evident in human cells may be unnoticed in murine cells, as our effect seen with FX on human SaOs-2 cells. This difference in mineralization between species could limit the utility of murine models for studying bone remodeling in hemophilia A.

Given that reduced BMD is a well-known and important comorbidity of hemophilia patients, these findings further raise concerns about the translatability of murine data. Bone remodeling is a highly regulated process that involves multiple signaling pathways [29,30], many of which may differ between humans and mice [18]. The lack of significant mineralization changes in murine cells suggests that murine models might not accurately reflect the causes for the bone-related complications seen in human hemophilia patients, particularly those resulting from chronic dysregulation of coagulation factors.

### 4.3. Cytokines and Osteoblast Function

Concerning the responses of osteoblasts to inflammatory cytokines, particularly TNF-α, significantly reduced cell viability in both murine and human osteoblasts, consistent with its known role as a potent inhibitor of osteoblast function and stimulator of osteoclast activity [31,32]. However, we observed opposite effects of TNF-α on mineralization: it stimulated mineralization in murine cells while inhibiting it in human cells. This suggests that TNF-α signaling functions differently across species, potentially contributing to the divergent outcomes in bone health observed in humans and mice under chronic inflammatory conditions [17,33]. Although our two-week assessments indicated similar trends to those observed at three weeks, the higher variability observed at two weeks—particularly in murine mineralization—suggests that responses may stabilize over extended culture periods, potentially reflecting progressive differentiation and maturation of osteoblasts.

Our findings align with the existing research on interspecies differences in cytokine signaling pathways, particularly in responses to IL-6 and TNF-α. Studies have shown that murine cytokines, including IL-6, often elicit distinct cellular responses due to species-specific pathways and receptor compatibilities, especially under stress or inflammatory conditions [34,35]. For instance, while human IL-6 can activate murine IL-6 receptors, murine IL-6 frequently lacks efficacy on human receptors [36], underscoring the challenges in using murine models to study cytokine effects relevant to human health. Additionally, recent research has highlighted that cytokine-driven pathways can vary significantly between species, impacting cell viability, metabolic processes, and inflammatory responses [34].

As seen in conditions like rheumatoid arthritis, chronic exposure to TNF-α is associated with reduced bone formation and increased bone resorption in humans [37]. The inhibitory effects of TNF-α on mineralization in human cells align with these observations. In contrast, the stimulation of mineralization in murine cells could indicate that murine osteoblasts respond differently to inflammatory cues, potentially due to differences in cytokine receptor expression or downstream signaling pathways. These differences are critical when studying bone loss in hemophilia A, where chronic inflammation plays a major role in disease progression.

### 4.4. Implications for Hemophilia A Research

Our findings have important implications for the study of bone health in hemophilia A. The distinct responses of murine and human osteoblasts to coagulation factors and cytokines suggest that murine models may not fully capture the complexity of bone remodeling in human hemophilia patients. Additionally, several human-modified coagulation factors, such as B-domain deleted FVIII, used in research and clinical applications, lack direct murine equivalents, complicating their study in murine systems.

Hemophilia A is characterized not only by deficiencies in coagulation factors but also by chronic joint inflammation, both of which significantly impact bone health [1,38]. The inability of in vitro models to mimic inflammatory responses, particularly in relation to bone metabolism, further limits their usefulness in preclinical research.

The lack of spontaneous joint bleeds in murine hemophilia in vivo models further complicates the study of bone health in these animals. In humans, recurrent joint bleeds and the resulting hemarthrosis are key contributors to bone loss, but this critical aspect of hemophilia is absent in mice [24,25]. As a result, murine models may underestimate the long-term effects of hemarthrosis on BMD in hemophilia patients.

### 4.5. Study Limitations and Future Directions

While this study provides valuable insights into the interspecies differences between murine and human osteoblasts, several limitations must be acknowledged. Our two-week data indicated variability in early mineralization responses, particularly in murine osteoblasts, which likely reflects the incomplete maturation of cells at this earlier time point. These findings reinforce our choice to prioritize three-week data for clearer, more consistent results. Future studies might consider focusing on longer time points or adjusting differentiation protocols to ensure more stable responses, especially in murine models, where osteoblast maturation may require extended culture periods.

We used human coagulation factors and cytokines to stimulate murine cells, which could introduce variability due to interspecies interactions. Structural and functional variations between murine and human coagulation factors are well-documented, particularly in factors such as FVIII and FIX, where limited homology in key domains may impact their function in the coagulation cascade [28]. These differences reinforce the translational limitations of murine models for human-specific research on coagulation factors, especially in conditions like hemophilia A. However, this approach is widely used in research without the full background of interspecies differences. Future studies could benefit from exploring species-specific factors when possible.

Additionally, our experiments were conducted under serum conditions, which could have influenced the results by providing growth factors that support cell viability and differentiation. Future research could explore the effects of these factors under serum-free or low-serum conditions to better isolate their direct effects on osteoblast function.

Our study also focused specifically on human SaOs-2 osteoblasts as a model to explore the direct effects of coagulation factors on bone-forming cells. The use of human osteoblasts in vitro offers a controlled environment to isolate and study specific cellular responses that are directly relevant to human bone biology, enabling a detailed analysis of how factors like FX affect osteoblast viability and mineralization. However, the in vitro system does not fully capture the complexity of the bone microenvironment found in vivo, where interactions between osteoblasts, osteoclasts, and other cell types are crucial for bone remodeling [29].

While our findings suggest that FVIII may not have a direct effect on osteoblast function, they should be interpreted in the context of a broader physiological system. The balance between bone formation and resorption, which is critical to understanding bone density in hemophilia A patients, involves additional factors that are beyond the scope of our current model. Future research should focus on incorporating co-culture models of osteoblasts and osteoclasts, or advanced 3D bone models, to provide a more complete picture of how FVIII deficiency impacts bone health. These approaches would build on the strengths of our current study, offering further insights into the cellular mechanisms that contribute to altered bone metabolism in hemophilia A.

## 5. Conclusions

This study highlights significant species-specific differences in the responses of murine and human osteoblasts to coagulation factors and inflammatory cytokines. These differences suggest major limitations of murine models when interpreting bone health in hemophilia A. While murine models have been instrumental in advancing our understanding of hemophilia, their ability to accurately reflect the complexities of human bone remodeling remains limited. Our findings emphasize the need for more physiologically relevant models to accurately examine the effects of coagulation and inflammation on bone health in hemophilia A.

Future research should focus on developing advanced humanized in vitro models that offer a more accurate representation of human bone biology. These could involve cultivating primary human osteoblasts from healthy donors or patients with hemophilia A. Co-culture experiments that incorporate the two main bone cell types, particularly in a 3D environment, could more accurately reflect the close interactions between osteoblasts and osteoclasts, thereby promoting the use of animal-free research. Such models have the potential to bridge the gap between basic research and clinical outcomes, providing new insights into the mechanisms driving bone loss in hemophilia and fostering the development of targeted therapeutic strategies.

## Data Availability

The original contributions presented in the study are included in the article/Supplementary Material, further inquiries can be directed to the corresponding author.

## Supplementary Materials

The following supporting information can be downloaded at: https://www.mdpi.com/2227-9059/12/12/2666/s1, Figure S1: The cell viability of murine MC3T3-E1 and human SaOs-2 osteoblasts in the presence of various coagulation factors; Figure S2. Representative images and photometric quantification of Alizarin red S staining in murine MC3T3-E1 and human SaOs-2 osteoblasts; Figure S3. The expression of Alizarin red S relative to cell viability in murine MC3T3-E1 and human SaOs-2 osteoblasts; Figure S4. The cell viability of murine MC3T3-E1 and human SaOs-2 osteoblasts in the presence of different cytokines; Figure S5. Representative images and photometric quantification of Alizarin red S staining in murine MC3T3-E1 and human SaOs-2 osteoblasts; Figure S6. The expression of Alizarin red S relative to cell viability in murine MC3T3-E1 and human SaOs-2 osteoblasts.
