# Supplementary material for: Murine vs. Human Osteoblast Responses to Coagulation and Inflammatory Factors: Reconsidering the Use of Animal Models in Hemophilia A Research
Source: Biomedicines. 2024 Nov 22;12(12):2666. doi: 10.3390/biomedicines12122666 (PMC11726731; doi:10.3390/biomedicines12122666)
Supplement: Supplementary file 1 [file biomedicines-12-02666-s001.zip › biomedicines-3261782-supplementary.pdf]

Murine vs. Human Osteoblast Responses to Coagulation and Inflammatory Factors: Reconsidering the Use of Animal Models in Hemophilia A Research

Supplementary Figures:

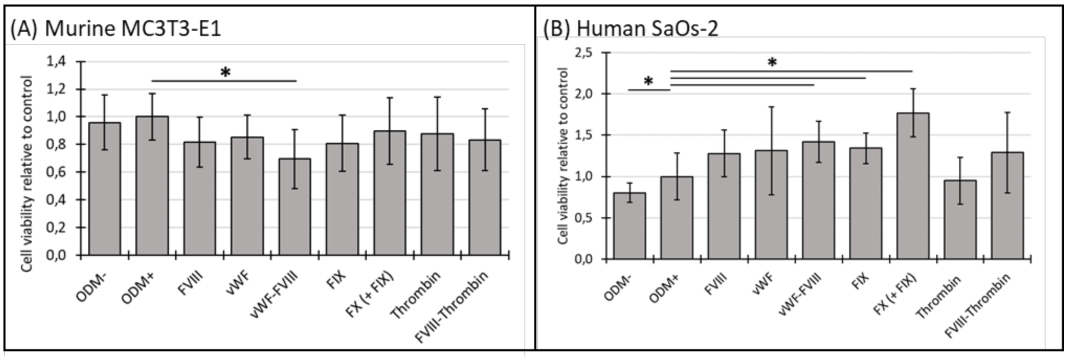

Figure S1: The cell viability of murine MC3T3-E1 and human SaOs-2 osteoblasts in the presence of various coagulation factors. (A) Murine MC3T3-E1 osteoblasts and (B) human SaOs-2 osteoblasts were incubated for two weeks in αMEM medium supplemented with ascorbic acid (1 mM), ß-glycerophosphate (8 mM), and calcium chloride (5 mM), referred to as osteoblast differentiation medium (ODM+). The negative control (ODM-) lacked these supplements. Cells were exposed to various coagulation factors (FVIII, vWF, vWF-FVIII, FIX, FX, thrombin, and FVIII-thrombin; 1 U/ml each). Cell viability was assessed relative to the ODM+ reference, set as 1. Data represent the means ± standard deviation of four independent assays performed in triplicates (n = 3), except for ODM+ reference and the negative control (ODM-) (n = 6). * indicates a significant difference ($p < 0.05$).

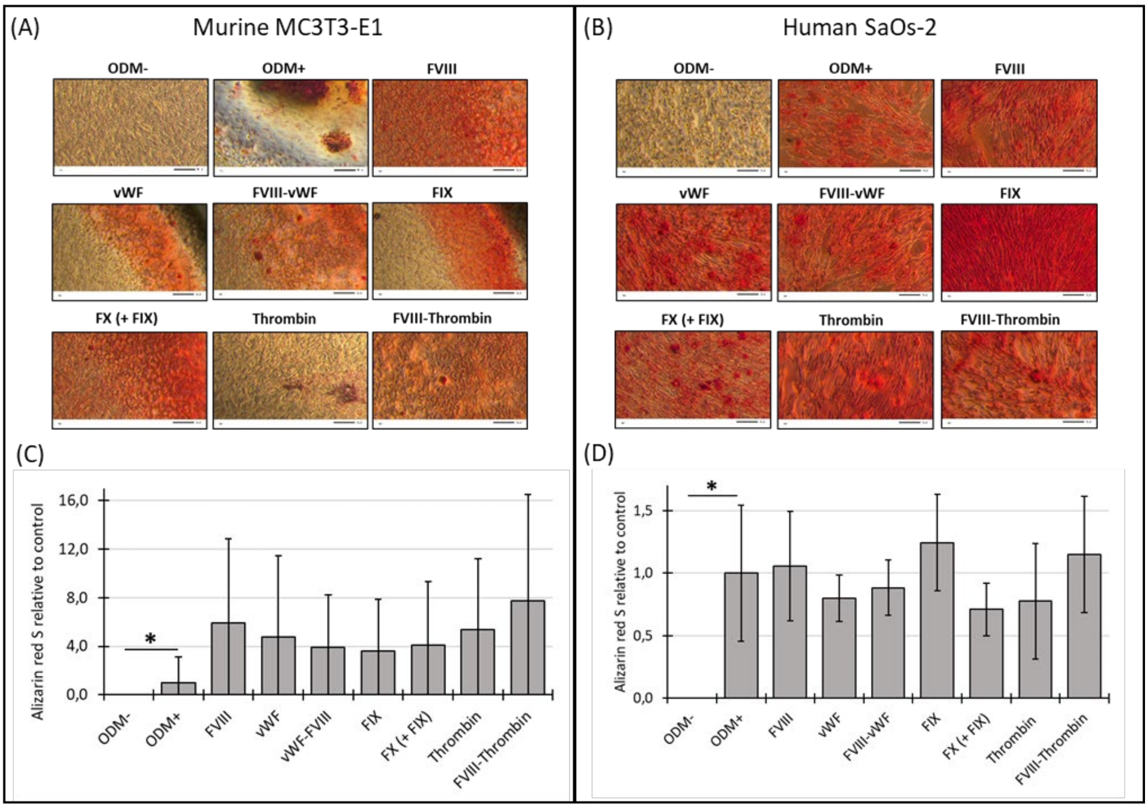

Figure S2. Representative images and photometric quantification of Alizarin red S staining in murine MC3T3-E1 and human SaOs-2 osteoblasts. Representative images of (A) murine MC3T3-E1 osteoblasts and (B) human SaOs-2 osteoblasts after two weeks of incubation in αMEM medium supplemented with ascorbic acid (1 mM), ß-glycerophosphate (8 mM), and calcium chloride (5 mM), referred to as osteoblast differentiation medium (ODM+). The negative control (ODM-) lacked these supplements. Cells were exposed to different coagulation factors (FVIII, vWF, vWF-FVIII, FIX, FX, thrombin, and FVIII-thrombin; 1 U/ml each). After incubation, cells were stained with Alizarin red S to assess mineralization, which was then quantified photometrically. Quantification results are shown for (C) murine and (D) human osteoblasts, with data normalized to the ODM+ reference. Data are presented as the means ± standard deviation of four independent assays performed in triplicates (n = 3), except for the ODM+ reference and the negative control (ODM-) (n = 6). * indicates a significant difference ($p < 0.05$). Images were captured using transmitted light microscopy at 20x magnification. Scale bar: 50 µm.

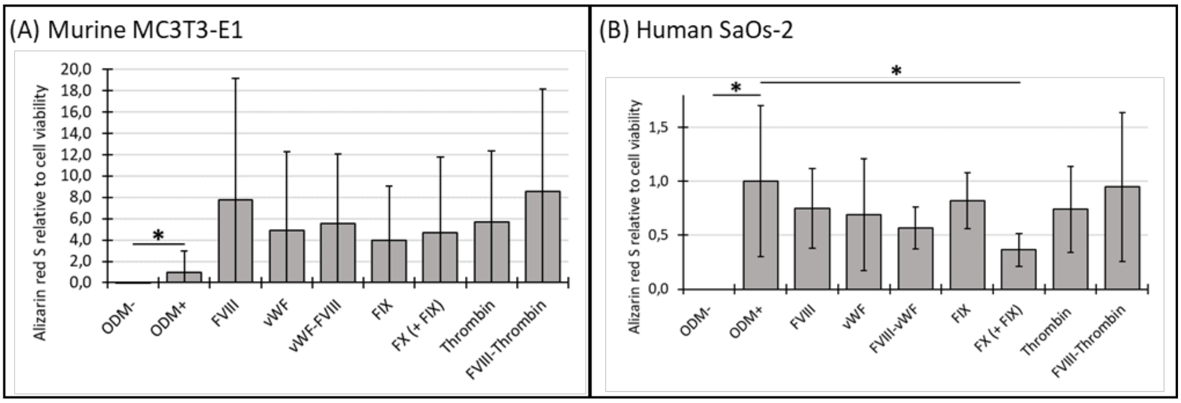

Figure S3. The expression of Alizarin red S relative to cell viability in murine MC3T3-E1 and human SaOs-2 osteoblasts. (A) Murine MC3T3-E1 osteoblasts and (B) human SaOs-2 osteoblasts were incubated for two weeks in αMEM medium supplemented with ascorbic acid (1 mM), ß-glycerophosphate (8 mM), and calcium chloride (5 mM), referred to as osteoblast differentiation medium (ODM+). The negative control (ODM-) lacked these supplements. Cells were exposed to various coagulation factors (FVIII, vWF, vWF-FVIII, FIX, FX, thrombin, and FVIII-thrombin; 1 U/ml each). The levels of Alizarin red S were quantified relative to cell viability and are expressed relative to the ODM+ reference, which was set as 1. Data represent the means ± standard deviation of four independent assays performed in triplicates (n = 3), except for the ODM+ reference and the negative control (ODM-) (n = 6). * indicates a significant difference ($p < 0.05$).

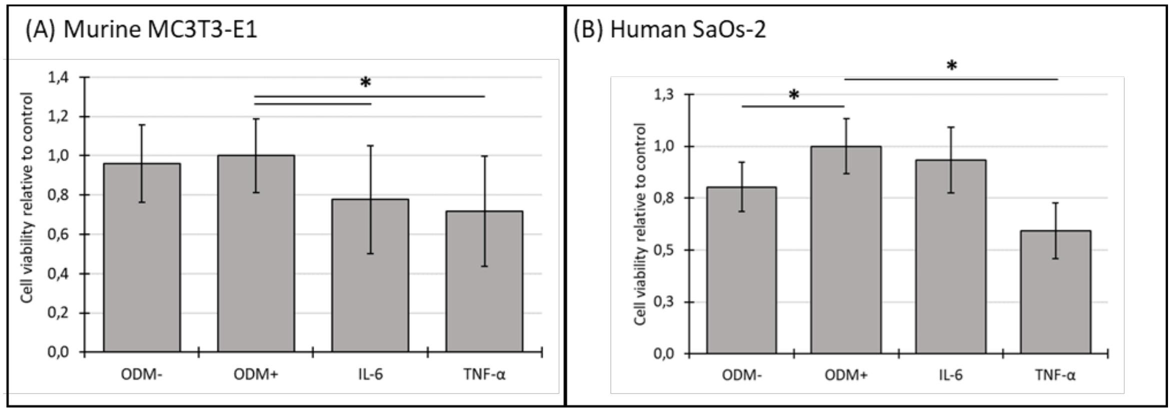

Figure S4. The cell viability of murine MC3T3-E1 and human SaOs-2 osteoblasts in the presence of different cytokines. (A) Murine MC3T3-E1 osteoblasts and (B) human SaOs-2 osteoblasts were incubated for two weeks in αMEM medium supplemented with ascorbic acid (1 mM), ß-glycerophosphate (8 mM), and calcium chloride (5 mM), referred to as osteoblast differentiation medium (ODM+). The negative control (ODM-) lacked these supplements. Cells were exposed to different cytokines (IL-6 and TNF-α; 50 ng/ml each). Cell viability was assessed relative to the ODM+ reference, which was set as 1. Data represent the means ± standard deviation of four independent assays performed in triplicates (n = 3), except for ODM+ reference and the negative control (ODM-) (n = 6). * indicates a significant difference ($p < 0.05$).

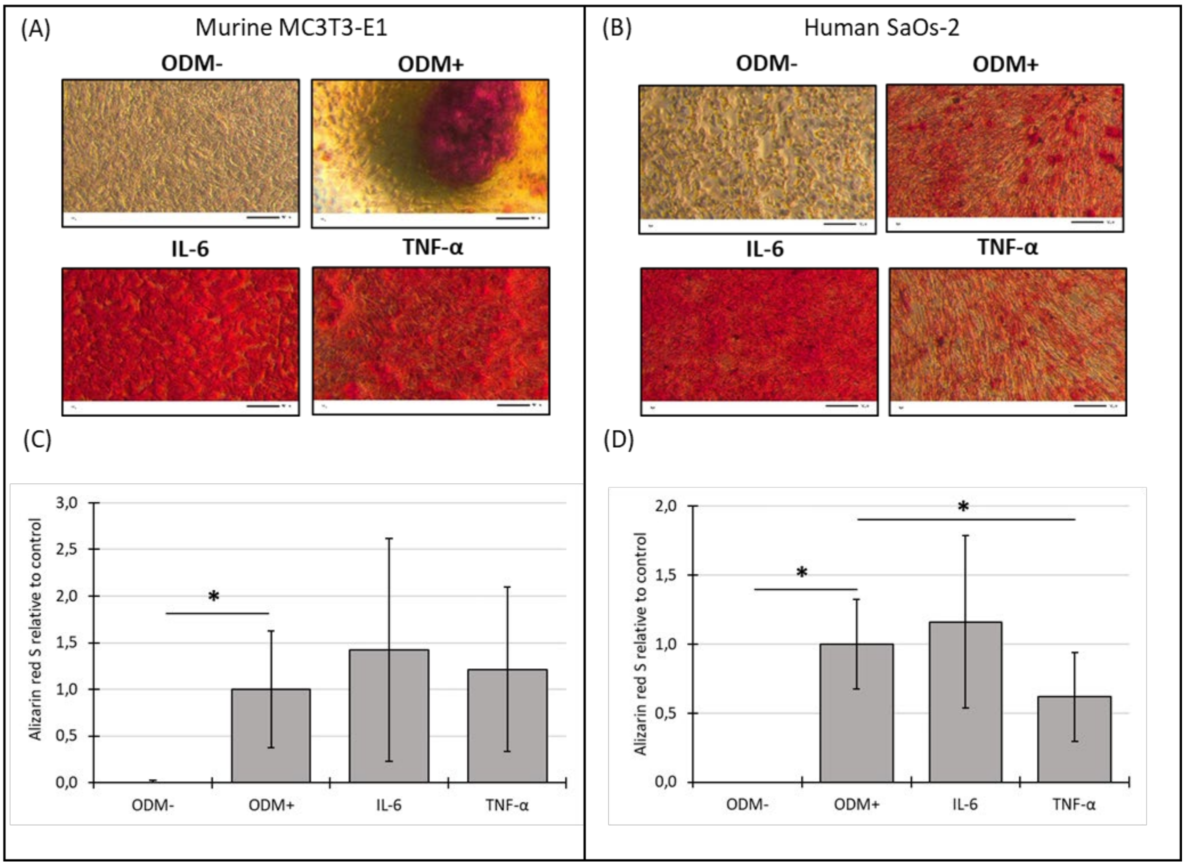

Figure S5. Representative images and photometric quantification of Alizarin red S staining in murine MC3T3-E1 and human SaOs-2 osteoblasts. Representative images of (A) murine MC3T3-E1 osteoblasts and (B) human SaOs-2 osteoblasts after two weeks of incubation in αMEM medium supplemented with ascorbic acid (1 mM), β-glycerophosphate (8 mM), and calcium chloride (5 mM), referred to as osteoblast differentiation medium (ODM+). The negative control (ODM-) lacked these supplements. Cells were exposed to different cytokines (IL-6 and TNF-α; 50 ng/ml each). After incubation, cells were stained with Alizarin red S to assess mineralization, which was then quantified photometrically. Quantification results are shown for (C) murine and (D) human osteoblasts, with data normalized to the ODM+ reference. Data are presented as the means ± standard deviation of four independent assays performed in triplicates (n = 3), except for the ODM+ reference and the negative control (ODM-) (n = 6). * indicates a significant difference ($p < 0.05$). Images were captured using transmitted light microscopy at 20x magnification. Scale bar: 50 µm.

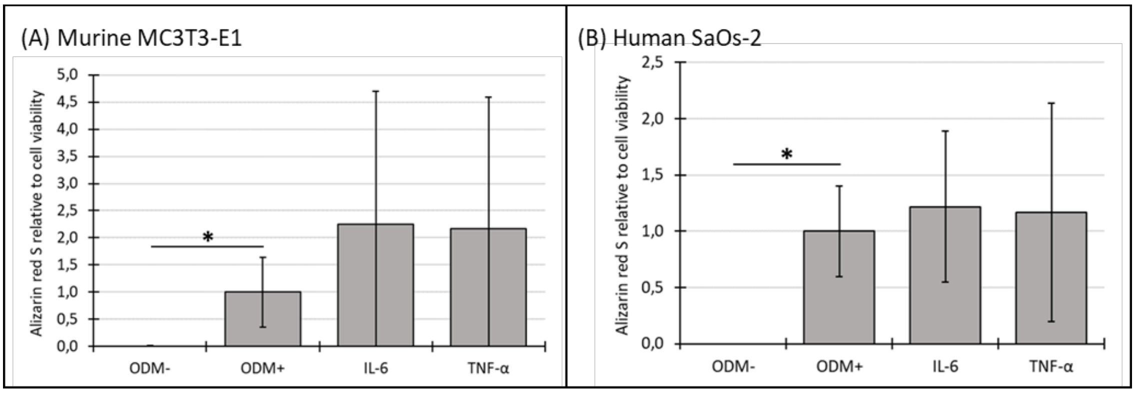

Figure S6. The expression of Alizarin red S relative to cell viability in murine MC3T3-E1 and human SaOs-2 osteoblasts. (A) Murine MC3T3-E1 osteoblasts and (B) human SaOs-2 osteoblasts were incubated for two weeks in αMEM medium supplemented with ascorbic acid (1 mM), ß-glycerophosphate (8 mM), and calcium chloride (5 mM), referred to as osteoblast differentiation medium (ODM+). The negative control (ODM-) lacked these supplements. Cells were exposed to different cytokines (IL-6 and TNF-α; 50 ng/ml each). The levels of Alizarin red S were quantified relative to the cell viability and are expressed relative to the ODM+ reference, which was set as 1. Values represent the means ± standard deviation of four independent assays performed in triplicates (n = 3), except for ODM+ reference and the negative control (ODM-) (n = 6). * indicates a significant difference ($p < 0.05$).
